# High-throughput base editing: a promising technology for precision medicine and drug discovery

**DOI:** 10.1038/s41392-021-00633-0

**Published:** 2021-06-04

**Authors:** Agapios Sachinidis

**Affiliations:** 1 Faculty of Medicine, Institute of Neurophysiology, University of Cologne, Cologne, Germany; 2 Center for Molecular Medicine Cologne (CMMC), University of Cologne, Cologne, Germany

**Keywords:** Molecular medicine, Genomics

Recently, two resource papers (Hanna et al.^1^ and Cuella-Martin et al.^2^) were published describing high-throughput large-scale functional assessment of tens of thousands of human single-nucleotide variants (SNVs) across multiple cell lines by base editing in single pooled screens.

RBase editing is a new CRISPR/Cas9 nuclease-based genome-editing tool. It is gaining its momentum in revolutionizing precision and personalized medicine by its ability to introduce point mutations (C → T or A → G) precisely at a specific endogenous DNA locus without the induction of double-strand breaks (DSBs), unlike the other CRISPR-based genome-editing methodologies^3^. Base editors (BEs) are composed of two components: a catalytically dead Cas9 (dCas9) or a nickase Cas9 (nCas9) fused to a deaminase and a single-guide RNA (sgRNA). The sgRNA guides to the desired locus and the dCas9 or nCas9 recognizes a specific sequence called Protospacer Adjacent Motif (PAM). Once the DNA unwinds upon sgRNA’s complementary pairing with the target DNA sequence, the deaminase converts bases in a specific DNA stretch of the protospacer located usually upstream of the PAM in the opposite DNA strand. There are two major classes of BEs; the cytosine BEs (CBEs) and adenine BEs (ABEs) that enable C → T and A → G conversions, respectively^3^. BE3, BE4 and ABE7.10 are the most commonly used BEs due to their improved efficiency and precision of editing. The editing window is at base positions 4–8 of the protospacer for BE3 and BE4 CBEs, and is at the 4–7 base positions for ABE7.10 (ABEs). Recent advances in the BEs’ specificity, precision and cellular delivery present exciting therapeutic opportunities for the treatment of a wide spectrum of genetic diseases. Around 60% of the human human pathogenic point mutations reported in *ClinVar* (https://www.ncbi.nlm.nih.gov/clinvar) database can be potentially corrected by BEs (Fig. 1).

**Fig. 1:**
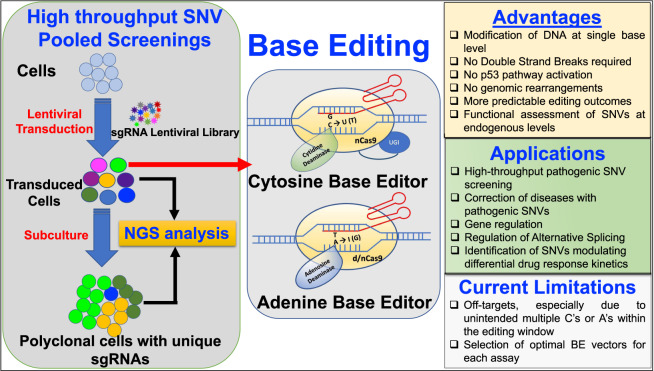
Schematic overview of BE based high-throughput SNV pooled screening methodology; Advantages, therapeutic applications and current limitations (NGS: Next Generation Sequencing; SNV: Single Nucleotide Variants)

At least three different cell lines were co-transduced with lentiviral BEs and sgRNA libraries targeting every NGG/PAM sequence (N: unknown base) within the coding regions of the 47 genes^1^ and 86 DNA damage response genes^2^, with a total library size of 12,141^1^ and ~37,000^2^ sgRNAs, respectively. The multiplicity of infection was around 0.4 so that 20–40% of the cells were transduced, resulting in a heterogeneous population of transduced (mostly one sgRNA/cell) and non-transduced cells. The transduced cells were positively selected by the respective antibiotic resistance conferred by the lentiviral vector. These cells were cultured for >10 days and analysed for their phenotypic changes such as their proliferative potential or cell death. The enriched sgRNA in the proliferating cells or depleted sgRNA in the cells undergoing cell death under different genotoxic stress conditions were analysed from genomic DNA isolated from the cells at day 0 and later points of the experiments by targeted deep genome sequencing by PCR amplifying the sgRNAs with the primers that bind to the regions flanking sgRNAs. These high-throughput functional screenings enabled accurate functional dissection of complex multidomain proteins, identification of different SNVs within the same gene offering growth advantage and disadvantages, and SNVs that confer resistance or sensitivity to different pharmacological drugs. These screenings distinguished pathogenic from benign mutations and identified hundreds of clinically relevant genetic variants exhibiting altered cellular growth and/or response to various genotoxic drugs. These works demonstrate the true potential of BEs–pooled screen approach in unravelling the functional consequences of causal variants identified by genome-wide association studies.

Application of the BEs and pooled screening approach in the induced pluripotent stem cells (iPSCs) disease modelling by generating isogenic iPSC lines offers an unprecedented opportunity in high-throughput drug discovery, safety pharmacology and elucidation of the pathological mechanisms underlying polygenic complex diseases involving the association of more than one genetic variants (schizophrenia and autism spectrum disorders), sporadic diseases for which the genetic causes were not known from the family history (Alzheimer’s disease), diseases with late-onset of phenotypic changes (Brugada syndrome and early repolarization syndrome) and diseases that involve the interaction of a multitude of phenotypic cells (amyotrophic lateral sclerosis). High-throughput screening with BEs^1,2^ combined with the human iPSC (hiPSC)-based disease modelling will revolutionize the personalized medicine by filling several gaps hindering the exploitation potential of the hiPSCs as a powerful therapeutic tool^4^. However, the low editing efficiency in iPSC should be improved.

Indeed, BEs has tremendous therapeutic potential for diseases associated with pathogenic SNVs due to their versatility to introduce multiple BEs in the same gene/gene families with minimal off-target effects compared to other pre-existing methodologies. As Bes do not require DSB induction, BEs will not evoke p53-mediated apoptosis and, hence, BEs are relatively safer genome-editing tools. BEs can be exploited to introduce de novo transcription factor-binding sites to overexpress transdifferentiating factors from endogenous promoters for direct conversion of blood cells or skin fibroblasts into clinically relevant phenotypic cells for cell replacement therapies of degenerative diseases such as heart failure and diabetes mellitus.

Nevertheless, both CBEs and ABEs could generate unintended base conversions at certain genomic loci and will reduce product purity by off-target effects. If multiple C or A are present in the same editing window, their unintended base editing will pose greater challenges in the therapeutic applications and needs to be thoroughly investigated. To ascertain whether a mutation is a dominant or recessive phenotype, it is very critical to distinguish between homozygous and heterozygous base editing.

The pooled screen with BEs will greatly benefit from the optimization of editing window, phenotypic resolution and editing specificity for every assay^2^. Also, the novel dual-function BEs that can simultaneously induce both A → G and C → T in the same target site could be a powerful approach for many therapeutic applications. Although both studies^1,2^ made significant progress in the CBE-based identification and correction of genes with pathological SNVs, the consequences of such corrections for the stability of the rest of the genome, which stands under ‘evolutionary pressure’ are unknown. Indeed, evolutionary perspective on human diseases should be taken into consideration when it comes to personalized medicine. In other words, corrections of genes that are responsible for a disease could modify the function of other gene/s due to the evolutionary memory of our cells^5^.

## References

[1] Hanna RE (2021). Massively parallel assessment of human variants with base editor screens. Cell.

[2] Cuella-Martin R (2021). Functional interrogation of DNA damage response variants with base editing screens. Cell.

[3] Molla KA, Yang Y (2019). CRISPR/Cas-mediated base editing: technical considerations and practical applications. Trends Biotechnol..

[4] Doss MX, Sachinidis A (2019). Current challenges of iPSC-based disease modeling and therapeutic implications. Cells.

[5] Benton ML (2021). The influence of evolutionary history on human health and disease. Nat. Rev. Genet..

